# Biomechanical and histological analyses of the fracture healing process after direct or prolonged reduction

**DOI:** 10.1186/s40001-018-0337-6

**Published:** 2018-09-04

**Authors:** Benedikt Peterburs, Anke Mittelstaedt, Philipp Haas, Maximilian Petri, Ralf Westphal, Christian Dullin, Stephan Sehmisch, Claudia Neunaber

**Affiliations:** 1 Hannover Medical School (MHH) -Trauma Department, Carl-Neuberg-Str. 1, 30625 Hannover, Germany; 2 Institute for Robotics and Process Control, Technical University Braunschweig, Mühlenpfordtstraße 23, 38106 Brunswick, Germany; 3 Department for Diagnostic and Interventional Radiology, University Medical Center Goettingen, Robert-Koch-Str. 40, 37075 Goettingen, Germany; 4 Italian Synchrotron Light Source ‚Elettra‘, SYRMEP Beamline, Trieste, Italy; 5 Department of Trauma Surgery, Orthopaedic and Reconstructive Surgery, University Medical Center Goettingen, Robert-Koch Str. 40, Goettingen, 37075 Germany

**Keywords:** Femoral fracture, Fracture healing, Surgical robot, Biomechanics, Histology, Rat, Bone turnover marker, Computer-assisted surgery

## Abstract

**Background:**

Reduction of femoral shaft fractures remains a challenging problem in orthopaedic surgery. Robot-assisted reduction might ease reduction and fracture treatment. However, the influence of different reduction pathways on patients’ physiology is not fully known yet. Therefore, the aim of this study was to examine the biomechanics and histology of fracture healing after direct and prolonged robot-assisted reduction in an in vivo rat model.

**Methods:**

144 male CD^®^ rats were randomly assigned to 12 groups. Each animal received an external fixator and an osteotomy on the left femoral shaft. On the fourth postoperative day, the 1× reduction groups received a single reduction maneuver, whereas the 10× reduction groups received the same reduction pathway with ten repetitions. The control groups did not undergo any reduction maneuvers. Animals were killed after 1, 2, 3 and 4 weeks, respectively, and the composition of the fracture gap was analyzed by µCT and non-decalcified histology. Biomechanical properties were investigated by a three-point bending test, and the bone turnover markers PINP, bCTx, OPG, sRANKL, TRACP-5b, BALP, and OT/BGP were measured.

**Results:**

One week after the reduction maneuver, µCT analysis showed a higher cortical bone volume in the 1× reduction group compared to the 10× reduction group. Biomechanically, the control group showed higher maximum force values measured by three-point bending test compared to both reduction groups. Furthermore, less collagen I formation was examined in the 10× reduction group compared to the control group after 1 week of fracture healing. PINP concentration was decreased in 10× reduction group after 1 week compared to control group. The same trend was seen after 3 weeks.

**Conclusion:**

A single reduction maneuver has a beneficial effect in the early phase of the fracture healing process compared to repeated reduction maneuvers. In the later phase of fracture healing, no differences were found between the groups.

## Background

Femoral shaft fractures commonly appear in multiple injured patients [1–4]. Severe soft tissue injuries are frequently seen due to high-energy trauma and can lead to blood loss of up to 1.5 L into the surrounding muscles [5]. Nonetheless, open fractures are only seen in 2–5% of femur fractures [5]. Manual reduction of femoral shaft fractures is cumbersome and afflicted with a variety of complications.

As robot-assisted devices aim to reach anatomical bone alignment, while X-ray exposure, soft tissue damage and surgical time are reduced, they could be a good tool to help physicians in the future.

Such an in vitro robot-assisted fracture reduction model has been described by Fuchtmeier [6], Koo [7] and Oszwald et al. [8]. However, the influence of different reduction repetitions on the bone healing process in vivo has not been examined yet. Accordingly, our study aimed to explore in a rat model the difference between direct reduction path and a reduction path with a prolonged reduction performed by a robot, to standardize the reduction pathway.

In a preliminary study, we established this in vivo rat model and analyzed the concentration of plasma cytokines and soft tissue damages of muscle biopsies after direct and prolonged reduction. The results showed that the pro-inflammatory cytokine IL-6 significantly increased 6 h after reduction in the prolonged reduction group compared to the direct reduction and control group. On the anti-inflammatory side, IL-10 showed a significant decrease in the prolonged reduction group compared to the direct reduction and control groups. Muscle biopsies showed a significant increase of pathological changes in both reduction groups and an increase in the severity of bleedings of the prolonged reduction group compared to the direct reduction and the control group [9].

In the present study, we investigated the composition of the fracture gap via µCT and the biomechanical stability of the injured bones after 1, 2, 3, and 4 weeks, respectively. Furthermore, we examined the fracture healing process by non-decalcified histology and measured the bone turnover markers type-I collagen N-terminal propeptide “PINP”, bone C-telopeptide of type-I collagen “bCTx”, osteoprotegerin “OPG”, soluble receptor activator of nuclear factor NF-kB ligand “sRANKL”, tartrate-resistant acid phosphatase “TRACP-5b”, human bone alkaline phosphatase “BALP” and osteocalcin/bone Gla protein “OT/BGP” after the reduction process at the same points in time.

## Methods

### Animal care

In total, 144 male CD^®^ rats weighing 350 ± 50 g with an age between 12 and 16 weeks, obtained from Charles River Laboratories (Charles River, Sulzfeld, Germany) were included in the study. The animals were held under pathogen-free conditions in the central animal facility of Hannover Medical School. Throughout the study, pellet chow and water were available ad libitum. Lighting was maintained on a 14-h light and 10-h dark cycle and at a temperature of 21 ± 2 °C.

### Group distribution

Rats were randomly assigned to 1 of 12 groups with 12 animals per group. In each group, six femora were used for biomechanics and six femora were used for histology. The group distribution can be seen in Table 1:

**Table 1:** Group distribution

Healing time (operation until euthanasia), days	Group	Number of animals
7	Control group (no reduction)	12
	1× reduction	12
	10× reduction	12
14	Control group (no reduction)	12
	1× reduction	12
	10× reduction	12
21	Control group (no reduction)	12
	1× reduction	12
	10× reduction	12
28	Control group (no reduction)	12
	1× reduction	12
	10× reduction	12

All groups received a fixation of the femur with an external fixator and subsequent osteotomy of the femoral shaft. The control group received no reduction process. The 1× reduction group received a single reduction attempt as described below and the 10× reduction group received the same reduction maneuver with ten repetitions.

### Fixation with external fixator and osteotomy

**Fig. 1:**
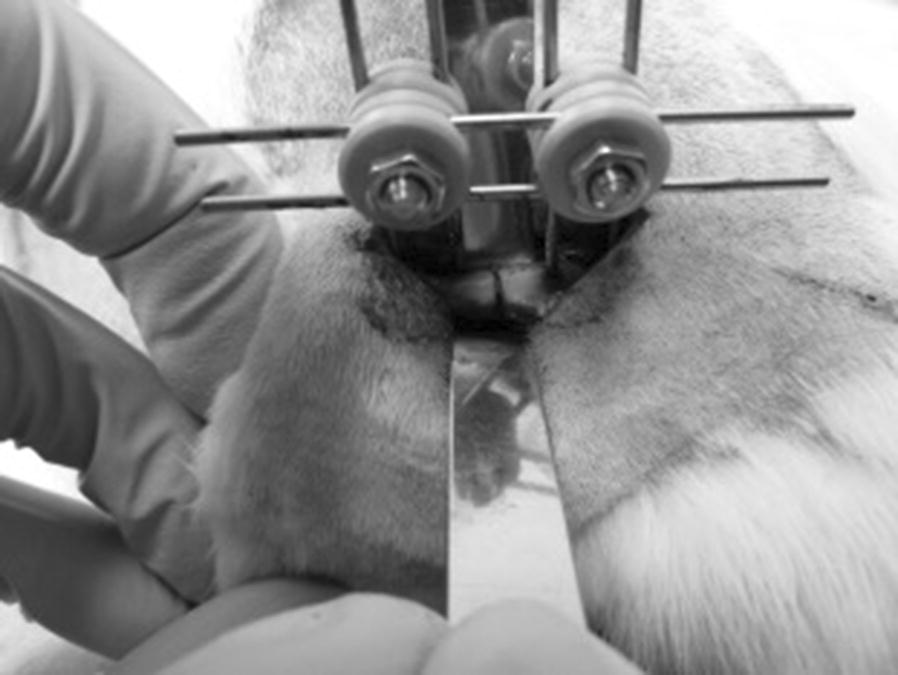
Lateral view onto the left hind limb of the rat with osteotomy and external fixateur placed in the femur

The surgical procedure was performed as previously published [9]. In brief, animals were anesthetized with Ketanest (60 mg/kg bodyweight) and Domitor (0.25 mg/kg bodyweight). Half of the dose was applied intraperitoneally for sedation and half was given subcutaneously for deep anesthesia. Rats were placed on a heating blanket and the left hind limb was shaved and disinfected. An incision was made from the knee to the hip joint. The fascia was opened and a blunt preparation of the femur was performed. A custom-made drillguide (Central Research Devices Service Unit of Hannover Medical School) with four holes was used for bicortical drilling with a 1.0-mm drill. A threaded pin (diameter 1.2 mm, stainless steel, Central Research Devices Service Unit of the Hannover Medical School) was inserted and bicortical placement was verified by palpation. All four pins were inserted and a custom-made external fixator (Central Research Devices Service Unit of Hannover Medical School) was attached to the pins. The fixator consisted of two dynamic fixation discs connected by two horizontal rods. After rigid fixation, an osteotomy was made between the two center pins using a Gigli saw (0.4 mm, RISytem, Davos, Switzerland) (Fig. 1). To ensure correct pin placement after osteotomy, a radiological image with a magnifying fluoroscope (Fluoroscan III 1996, Hologic Inc., Marlborough, USA) was taken. After rinsing with 0.9% NaCl, wound closure in two layers (fascia: Prolene^®^ 5/0, Ethicon, Norderstedt, Germany, skin: Prolene^®^ 3/0, Ethicon, Norderstedt, Germany) was performed.

The animals had a resting period of 3 days after surgery to eliminate the impact of the surgical procedure. Animals were visited daily and postoperative pain was reduced for 4 days postop by adding Turbogesic (1 mg/kg bodyweight) to the drinking water.

### Robot-assisted reduction of femoral fracture

The reduction maneuver was performed according to the previous published study [9]. In brief, an industrial robot (Stäubli RX 90, Stäubli Tec System, Faverges, France) with its standard robot control unit (CS7B) was used for the reduction procedure. The robot was controlled with a Windows PC and a self-made control software written in C++. As already described by Oszwald et al. [8], the aim of the reduction procedure was not to exceed realistic tensions and forces compared to those in the reduction procedure performed in humans. Therefore, we chose a maximum distraction up to one shaft diameter. The reduction process included a distraction of 2 mm, a vertical displacement of 5 mm upwards and 10 mm downwards, and a final movement backwards to the starting position.

On the fourth day after receiving the external fixator, the animals were anesthetized as described above. The two distal pins of the external fixator were attached to the robot’s hand and the two proximal pins to a fixation device mounted on the table. A torque sensor (FT Delta SI-660-60; Schunk, Lauffen, Germany) monitored the working load. Before reduction, the two horizontal rods of the external fixator were removed. The single reduction group received the reduction path once, the prolonged reduction group ten times, and the control group received no reduction. Once back in the final position, the two horizontal rods were reinserted for rigid stabilization and again a fluoroscopic image was taken to ensure correct alignment of the fracture.

### Killing and sampling

Blood was collected by retrobulbar sinus puncture before starting the reduction process after 3 days (0 h). To obtain all other samples, animals were killed via cardiac puncture under general anesthesia according to their group distribution 1, 2, 3 or 4 weeks after the reduction process. Blood was centrifuged at 7000 rpm (Heraeus Instruments 400R, Hanau, Germany) at room temperature and the plasma supernatant was transferred into a fresh tube, snap-frozen and stored at −80 °C until ELISA was performed.

Afterwards, the hind leg was removed by cutting the soft tissue and dislocating the hip joint while always paying close attention not to tamper with the external fixation. After exposing the femur, the horizontal and the fixation rods were removed and the samples were stored in a − 20 °C freezer or were bedded in formalin.

### Micro-computed tomography (µCT)

After thawing, the samples were scanned in a Micro-CT (eXplore Locus SP, GE Health Care, USA) which can be used for non-destructive ex vivo investigations. The region of interest was set at 2.2 mm proximally and distally around the fracture gap. The parameter settings were tube voltage = 72 kV, tube current = 90 µA, number of views = 900, exposure time 1600 ms and effective pixel size = 0.029 mm. Due to a calibration standard incorporated in the restraining container, the scans were comparable regarding their X-ray attenuation (respectively, their gray value, i.e. density).

Analysis was performed with the help of the 3D OsteoAnalyze program of our cooperation partners from the Small Animal Imaging Center of the University Medical Center, Göttingen, with which we were able to differentiate between soft callus, hard callus and cortical bone and quantify these parameters using a gray value histogram.

### Three-point bending test

Three-point bending test was performed using a material testing machine (Typ145660 Z020/TND Zwick/Roell, Ulm, Germany) with a custom-made mounting plate. The three bearings of the loading consisted of the head of femur, which was rested in an immersion (4 mm diameter) and the two femur condyles. To prevent the distal part of the femur to move sideways and to achieve a tight and central fit of the bone, to vertical adjusting bolds (5 mm diameter) mounted distally of the femur could be moved and fixated on an underlying rail. Once the bone was sufficiently positioned on its head of femur and the condyles, a roller stamp was driven down till the primary strength of 1 N was reached. After a final visual check of the correct femur position, the roller stamp was driven further at a constant velocity of 5 mm/min. The pressure was monitored every 0.001 mm until the bone broke. By monitoring the pressure and plotting it against the distance covered in a load–distance diagram, we were able to calculate the elasticity (N/mm), maximum force (N), breaking force (N) and yield strength (N).

### ELISAs

The ELISAs were performed according to the manufacturer’s instructions with plasma samples to detect the alteration of the bone turnover markers type-I collagen N-terminal propeptide “PINP” (Uscn Life Science Inc., Wuhan, China, Catalog no. E90957Ra), bone C-telopeptide of type-I collagen “bCTx” (Uscn Life Science Inc., Wuhan, China, Catalog no. Catalog no. E90892Ra), Osteoprotegerin “OPG” (CUABIO, Baltimore, USA, Catalog no. CSB-E07404r), soluble receptor activator of nuclear factor NF-kB ligand “sRANKL” (CUABIO, Baltimore, USA, Catalog no. CSB-E05126r), tartrate-resistant acid phosphatase “TRACP-5b” (CUABIO, Baltimore, USA, Catalog no. CSB-E08491r), Human bone alkaline phosphatase “BALP” (CUABIO, Baltimore, USA, Catalog No. CSB-E11865r) and Osteocalcin/Bone Gla Protein “OT/BGP” (CUABIO, Baltimore, USA, Catalog No. CSB-E05129r).

### Non-decalcified histology

Histological slides were performed using Technovit^®^ 9100 (Heraeus Kulzer, Wehrheim, Germany) according to the manufacturer’s instructions. This is a polymerization system based on methyl methacrylate (MMA), which was developed for embedding mineralized tissue for use in the light microscopy. We used a microtome (Leica RM2165; Techno-Med GmbH, Bielefeld) for cutting 5-μm slices out of the Technovit-blocks. Afterwards, the slices were put on object slides. Before staining could be performed the slides had to be dried at 60 °C for at least 7 days. Staining for detection of the different tissues in the callus was performed using pentachrome staining. Therefore, the slides were incubated in a descending alcohol line and afterwards stained with alcian blue, Weigert’s iron hematoxylin, Brilliant crocein R–Acid Fuchsin and Safran du Gatinais.

Analysis of the callus was done using a histological score based on the work of Goldberg and Oryan et al. [10, 11] (Table 2).

**Table 2:** Histological score

State of the callus formation	Points
Fracture gap not closed or closed with fibrous tissue	0
Fracture gap closed with cartilage	1
25% of the fracture gap closed with bone	2
50% of the fracture gap closed with bone	3
75% of the fracture gap closed with bone	4
100% of the fracture gap closed with bone	5

### Statistical analysis

Statistical analysis was performed after consultation of the Institute of Biometrie of the MHH using SPSS 21 (IBM, New York, USA). All data were non-parametric. Therefore, comparison between groups was performed using non-parametric Kruskal–Wallis test. Results were considered statistically significant at a probability of 0.05 or less. Results are expressed as median ± 25% percentile. The primary experimental outcomes were the biomechanical analysis of the cortical bone volume (mm^3^) and the maximum force (%). The secondary experimental outcomes were the plasma concentration of PINP (pg/ml) and the evaluation of the histological scores.

## Results

### Cortical bone volume

**Fig. 2:**
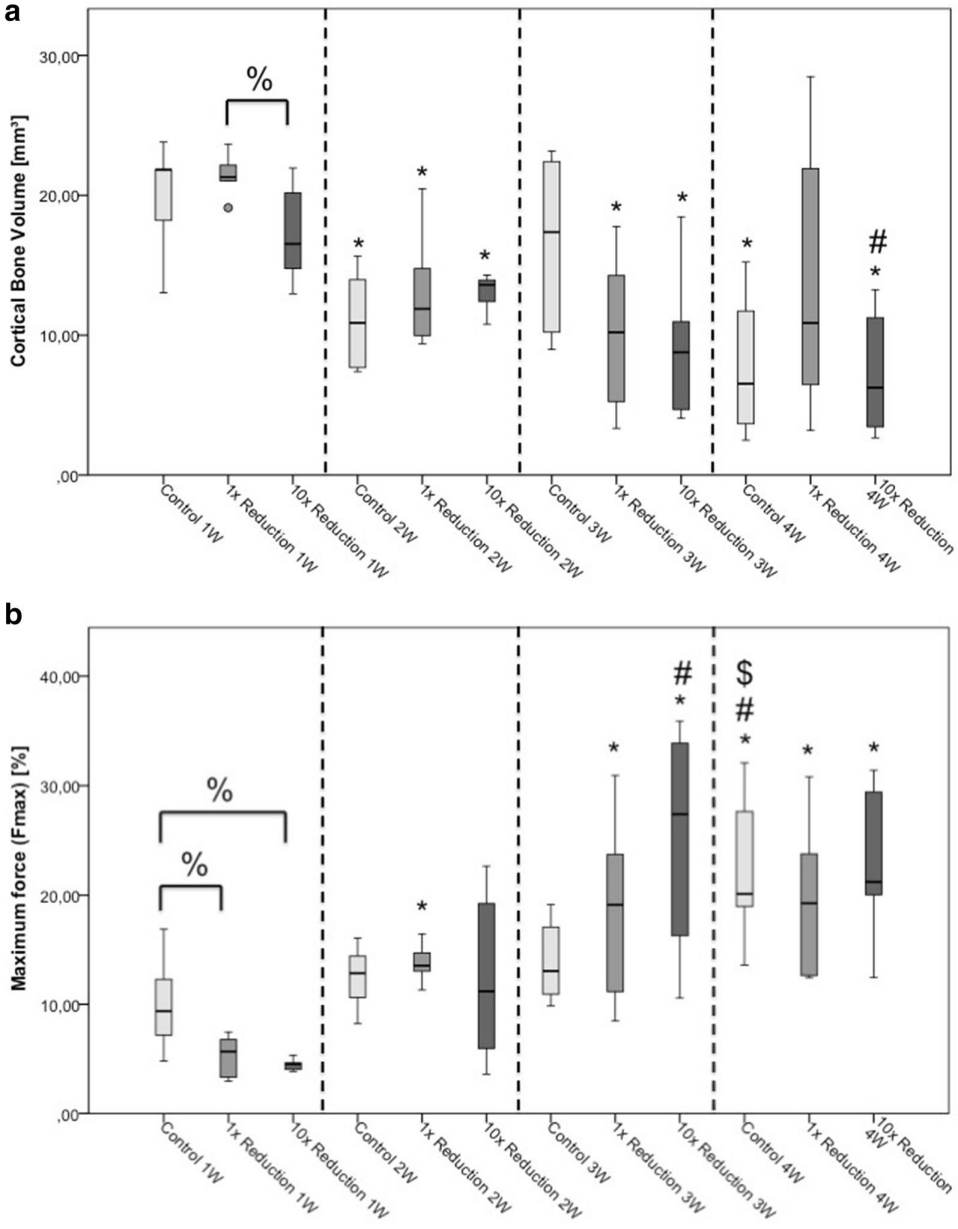
Cortical bone volume (**a**) and maximum force (**b**).*p ≤ 0.05 compared to week one of the same reduction maneuver; ^#^*p* ≤ 0.05 compared to week two of the same reduction maneuver; ^$^*p* ≤ 0.05 compared to week three of the same reduction maneuver; ^%^*p* ≤ 0.05 compared to indicated group

The amount of cortical bone compared to the overall bone volume was significantly higher in the 1× reduction group compared to the 10× reduction group 1 week after the reduction procedure (*p* = 0.045; Fig. 2a). Moreover, a significant decline in cortical bone volume in almost all study groups was observed compared to the first week, respectively (control 1 W vs. control 2 W: *p* = 0.018; control 1 W vs. control 4 W: *p* = 0.027; 1× reduction 1 W vs. 1× reduction 2 W: *p* = 0.011, 1× reduction 1 W vs. 1× reduction 3 W: *p* = 0.006, 10× reduction 1 W vs. 10× reduction 2 W: *p* = 0.025; 10× reduction 1 W vs. 10× reduction 3 W: *p* = 0.025, 10× reduction 1 W vs. 10× reduction 4 W: *p* = 0.006, 10× reduction 2 W vs. 10× reduction 4 W: *p* = 0.016; Fig. 2a).

### Maximum force

The obtained maximum force increased over the observation period in all groups. A significantly lower maximum force between the 1× reduction group (*p* = 0.037; Fig. 2b), as well as the 10× reduction group (*p* = 0.011; Fig. 2b) compared to the control group was observed 1 week after the reduction process. Moreover, it was evident that after 4 weeks the maximum force in the control group was significantly higher than in all previous weeks (control 1 W vs. control 4 W: *p* = 0.006; control 2 W vs. control 4 W: *p* = 0.016; control 3 W vs. control 4 W: *p* = 0.028; Fig. 2b), whereas the other groups were only significantly higher compared to their first-week peers (1× reduction 1 W vs. 1× reduction 2 W: *p* = 0.006, 1× reduction 1 W vs. 1× reduction 3 W: *p* = 0.004, 1× reduction 1 W vs. 1× reduction 4 W: *p* = 0.004, 10× reduction 1 W vs. 10× reduction 3 W: *p* = 0.006; 10× reduction 2 W vs. 10× reduction 3 W: *p* = 0.037, 10× reduction 1 W vs. 10× reduction 4 W: *p* = 0.009; Fig. 2b).

### Bone turnover marker

Analysis of the concentration of soluble receptor activator of NF-kB ligand (sRANKL), osteocalcin (OT/BGP), osteoprotegerin (OPG), beta-crossLAPS (bCTX), bone-specific alkaline phosphatase (BALP) and tartrate-resistant acid phosphatase 5b (TRACP-5b) showed no significant differences between the reduction groups at any point in time. Therefore, these results are not discussed further.

**Fig. 3:**
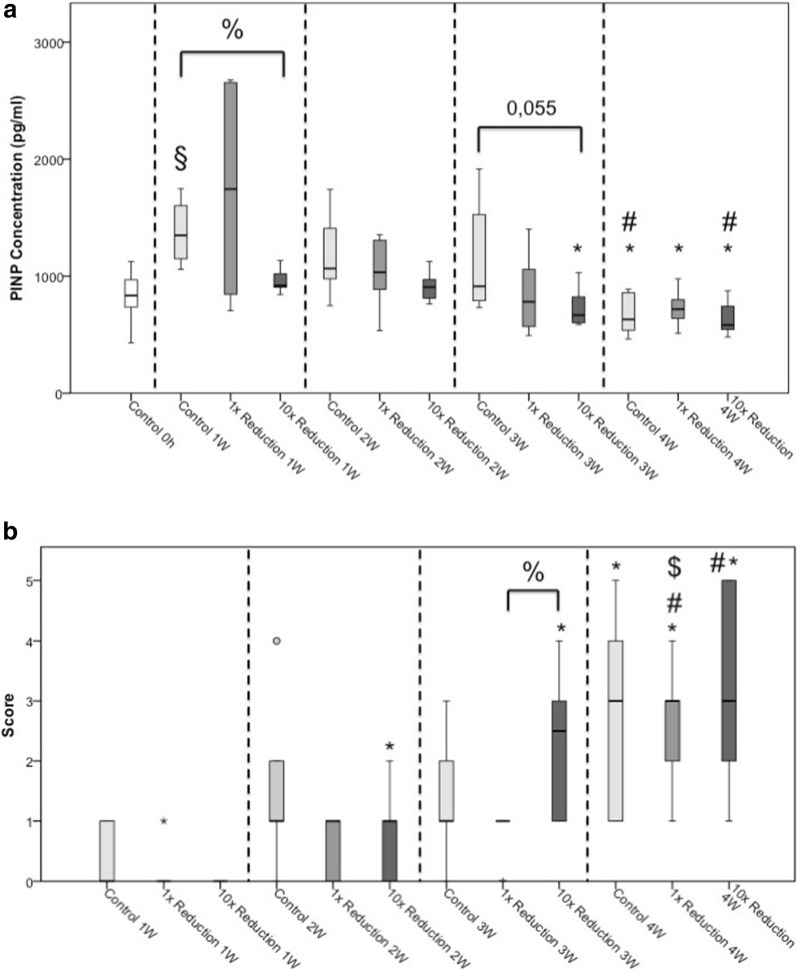
**a** Bone formation marker PINP. **b** Histological score. *p ≤ 0.05 compared to week one of the same reduction maneuver; ^#^*p* ≤ 0.05 compared to week two of the same reduction maneuver; ^$^*p* ≤ 0.05 compared to week three of the same reduction maneuver; ^%^*p* ≤ 0.05 compared to indicated group. ^§^*p* ≤ 0.05 compared to the respective 0 h group

The concentration of type I collagen N-terminal propeptide (PINP) showed a significant rise in the control group in the first week compared to control 0 h, which was taken immediately before reduction was performed (*p* = 0.014, Fig. 3a). Furthermore, less PINP concentration was measured in the 10× reduction group compared to control group (*p* = 0.027, Fig. 3a). After 3 weeks, a lower PINP concentration is indicated in the 10× reduction group compared to the control group (*p* = 0.055, Fig. 3a).

Besides, a significant decline of the bone formation marker PINP was observed between the following groups 1 week after the reduction process compared to the same groups 4 weeks after the reduction process: control 1 W vs. control 4 W: *p* = 0.011; 1× reduction 1 W vs. 1× reduction 4 W: *p* = 0.045; 10× reduction 1 W vs. 10× reduction 4 W: *p* = 0.011 (Fig. 3a). Additionally, the control group as well as the 10× reduction group showed significant lower PINP concentrations 4 weeks after the reduction process compared to the respective 2-week groups (control 2 W vs. control 4 W: *p* = 0.01; 10× reduction 2 W vs. 10× reduction 4 W: *p* = 0.018; Fig. 3a).

### Histological score

In general, we noticed a rise in the histology score over the observation period of 4 weeks according to the physiological fracture healing (control 1 W vs. control 4 W: *p* = 0.014; 1× reduction 1 W vs. 1× reduction 3 W: *p* = 0.027, 1× reduction 1 W vs. 1× reduction 4 W: *p* = 0.003, 1× reduction 2 W vs. 1× reduction 4 W: *p* = 0.011, 1× reduction 3 W vs. 1× reduction 4 W: *p* = 0.008; 10× reduction 1 W vs. 10× reduction 2 W: *p* = 0.021, 10× reduction 1 W vs. 10× reduction 3 W: *p* = 0.002, 10× reduction 1 W vs. 10× reduction 4 W: *p* = 0.003, 10× reduction 2 W vs. 10× reduction 3 W: *p* = 0.037, 10× reduction 2 W vs. 10× reduction 4 W: *p* = 0.024; Fig. 3b).

In the reduction groups, a delayed formation of cartilage was observed, which started after 2 weeks compared to the control group, in which the formation already started after 1 week. At that time, we only found small isles of cartilage in the reduction groups, but by far not enough to close the fracture gap. Also, in the second week, the reduction groups only achieve an average score of one point whereas the control group gains an average score of two points. All these observations are without statistical significance.

However, in the third week, the 10× reduction group achieves a median of three points compared to the single reduction group with a median score of one point (*p* = 0.02, Fig. 3b). Overall, despite the delay in the beginning, the 10× reduction group achieves the best histological score after 4 weeks. Two out of five animals achieve the maximum score of 5 points. In the comparable single reduction group, no animal achieves the maximum score and in the control group only one out of six animals, but none of these results are statistically significant.

## Discussion

The most important finding of our study was that a single reduction maneuver has a beneficial effect in the early phase of the fracture healing process compared to repeated reduction maneuvers. In the later phase of fracture healing, no differences were found between the groups. One week after reduction, there was a higher cortical bone volume in the 1× reduction group compared to the 10× reduction group and higher values of the maximum force in the control group compared to both reduction groups. Furthermore, less collagen I formation was observed in the 10× reduction group compared to the control group after 1 week. In the third week, the 10× reduction group achieved a significantly better histological score compared to the 1× reduction group.

To best of our knowledge, this is the first study to investigate the impact of different reduction maneuvers onto fracture healing. Therefore, we were not able to compare our findings with those of other authors. In contrast to our study, the variable in other studies, e.g. the rigidity of the fixation, was present in these studies throughout the whole time of fracture healing.

The observations in the first week could be caused by an altered cytokine composition in the early phase of the fracture healing process triggered by the prolonged reduction process. A fracture leads to the rupture of blood vessels and destroys the surrounding soft tissue. This destruction initiates the inflammatory cascade and fracture healing [12]. Subsequent vasodilation leads to exudation of plasma and leucocytes [13, 14]. The newly formed fracture hematoma contains peripheral blood-derived inflammatory cells [15], pro- and anti-inflammatory cytokines and mesenchymal stem cells [12, 16]. This inflammatory phase lasts about 7 days in rats [17].

In our previous study, we analyzed muscle biopsies and cytokine concentrations after direct and prolonged reduction in the same animal model. In this study, we observed a more severe bleeding in the 10× reduction group compared to the 1× reduction and the control group. Furthermore, the concentration of the pro-inflammatory interleukin-6 (IL-6) was significantly increased and the concentration of the anti-inflammatory IL-10 was significantly decreased in the 10× reduction group after 6 h compared to the 1× reduction group [9].

Through these changes in the cytokine composition, the early inflammatory phase of the fracture healing process is prolonged, which could lead to delayed fracture healing.

Another study further supports the suggestion that a prolonged and higher inflammation during the fracture healing process could be the reason for the decreased cortical bone volume and maximum force. They showed that the induction of inflammation by the administration of lipopolysaccharides after a mid-diaphyseal osteotomy of the femoral bone and subsequent nailing led to a hypertrophic and less mineralized callus after 6 weeks [18]. In one group, lipopolysaccharides were applied intraperitoneally (systemically), whereas in another group they were administered locally at the fracture site. Both groups had significantly lower values in callus area, bone mineral density and bone mineral content of the callus as well as fracture energy and bending moment compared to the control group.

Consistent to our findings is the study of Mølster et al. [19], who investigated the stabilization of a femoral shaft fracture in rats by intramedullary nailing. The focus of the study was on the fixation of the nail, which was either not at all, only distally, or locked at both ends, and thus produced a gradual rotational instability at the fracture site. It has been found that fractures, which were particularly unstable, in the parameter of strength exhibited the lowest values after 4 weeks of bone healing. Although instability at the fracture site is not directly comparable to our investigations, this study found similar results regarding the biomechanical evaluation. The work of Utvåg et al. [20], in which the group distribution was also divided by the blocking of the intramedullary nail in different groups of rotatory stability at the fracture site, showed no difference in the need for a re-fracture force after 6 weeks.

Furthermore, it was shown by Wang et al. [21] that when comparing intramedullary nailing with an elastic nail after osteotomy in a rabbits’ femur, the absorbable force before breaking the healed fracture was found to be the highest in the group stabilized with a rigid intramedullary nail. This advantage, however, was only traceable for the initial phase of healing, namely the period of 4 weeks. Then there was a superiority of less rigid materials. In our study, however, only an advantage in the control group could be recognized in the all-time rigidly fixed femur fracture. Furthermore, this advantage was not measured in the fourth week after the fracture, but within the first week of healing. However, the results found in this rabbit model, especially when regarding the temporal aspects of the healing process, are certainly limited in their comparability.

The bone formation marker PINP (type I collagen N-terminal propeptide) shows a lower concentration in the fourth compared to the first week in all three groups, which demonstrates a decline of the collagen I formation in between the first 4 weeks after fracture. Conspicuously, there is a significant rise between the zero sample, which was collected right before reduction, and the control group of the first week. This rise is not apparent in the reduction groups. It could be prevented by the second trauma through reduction process. Supporting this theory, there are significant lower PINP concentrations in the 10× reduction group after the first and the third weeks compared to the control group. But we are not able to show this significant difference in the 1× reduction because of the big range. In humans, there are no differences of PINP concentrations shown between patients with normal or delayed fracture healing so far [22]. However, for all of our tested bone turnover markers there are prior studies regarding the trend of these markers after fracture [23–29]. In terms of the bone resorption marker bCTx, Moghaddam et al. [22] observed a significant decrease of the absolute bCTx values during the first week in case of delayed fracture healing. Therefore, we expected a significant decrease in the 10× reduction group compared to the single reduction group, which we could not show. Since sRANKL and OPG are regulated by cytokines (e.g. IL-6) [30], we anticipated according to our prior study [9] significant changes in between the three different reduction groups. As already mentioned above, we were not able to show any significant changes of the bone turnover markers between the reduction groups at any point in time.

Regarding the histological investigation, we see a rising score according to the physiological fracture healing over time [2, 11, 13]. Also apparent is a delayed rise of the score in both reduction groups. As already described above, the second trauma also leads to a new inflammatory reaction with another invasion of inflammatory cells and releasing of inflammatory mediators [12]. This acute inflammatory response was artificially extended and newly aroused in the rats that received reduction.

Interestingly, after 3 weeks the 10× reduction group achieves a significant better score than the group of the single reduction attempt. Grundnes et al. [31] showed that the fracture healing is impaired if the fracture hematoma is removed 2–4 days after fracture in rats. Therefore, the hematoma seems to have an important role in fracture healing. It is even possible that a bigger or newly aroused fracture hematoma in terms of a prolonged reduction could have a positive effect on the healing process.

There were certain limitations that might have influenced the outcome of our study. The fixation devices in our study were custom-made. Even though we put a focus onto rigid fixation, there are certain limitations to the method used. First, there could have been movement in the connection between the bone and the fixation pin. Second, 4 days after the operation, the horizontal rods had to be removed and reassembled to connect the fixation pins to the robot, creating another possibility for fracture instability.

Moreover, there was a broader scattering of the results than we expected, possibly due to the fact that this was the first long-term in vivo study, whereas previous studies were for a shorter period or in vitro. It might be possible that the maximum distraction of one shaft diameter and only ten repetitions were inadequate to demonstrate the differences between the groups. The duration of the reposition process could have imposed a limiting factor as well. Manual reposition of a femoral fracture lasts between 6 and 28 min [32], whereas the 10× reduction process in our study took 36 s.

Furthermore, the osteotomy gap had only a size of 0.4 mm and the pins were applied before osteotomy to perform a standardized osteotomy. Therefore, only a small fracture gap and no malreduction were analyzed in our study setting. In a planned future animal model, we would like to create a more complex fracture closer to reality than a horizontal osteotomy such as used here. Pin insertion would be performed after the fracture in this case. We would then like to perform a robot-assisted reduction according to a previously computer-programmed pathway. This future model would also allow for analysis of a malreduction group.

## Conclusion

A single reduction maneuver has a beneficial effect in the early phase of the fracture healing process compared to repeated reduction maneuvers. In the later phase of fracture healing, no differences were found between the groups.
